# Stir bar sorptive-dispersive microextraction by a poly(methacrylic acid-co-ethylene glycol dimethacrylate)-based magnetic sorbent for the determination of tricyclic antidepressants and their main active metabolites in human urine

**DOI:** 10.1007/s00604-021-05156-7

**Published:** 2022-01-08

**Authors:** Víctor Vállez-Gomis, Sara Exojo-Trujillo, Juan L. Benedé, Alberto Chisvert, Amparo Salvador

**Affiliations:** GICAPC Research Group, Department of Analytical Chemistry, University of Valencia, 46100 Burjassot, Valencia, Spain

**Keywords:** Active metabolites, Human urine, Magnetic nanoparticles, Polymeric sorbent, Stir bar sorptive-dispersive microextraction, Tricyclic antidepressants

## Abstract

A poly(methacrylic acid-co-ethylene glycol dimethacrylate)-based magnetic sorbent was used for the rapid and sensitive determination of tricyclic antidepressants and their main active metabolites in human urine. This material was characterized by magnetism measurements, zeta potential, scanning electron microscopy, nitrogen adsorption–desorption isotherms, and thermogravimetric analysis. The proposed analytical method is based on stir bar sorptive-dispersive microextraction (SBSDME) followed by liquid chromatography–tandem mass spectrometry. The main parameters involved in the extraction step were optimized by using the response surface methodology as a multivariate optimization method, whereas a univariate approach was employed to study the desorption parameters. Under the optimized conditions, the proposed method was properly validated showing good linearity (at least up to 50 ng mL^−1^) and enrichment factors (13–22), limits of detection and quantification in the low ng L^−1^ range (1.4–7.0 ng L^−1^), and good intra- and inter-day repeatability (relative standard deviations below 15%). Matrix effects were observed for the direct analysis of urine samples, but they were negligible when a 1:1 v/v dilution with deionized water was performed. Finally, the method was successfully applied to human urine samples from three volunteers, one of them consuming a prescribed drug for depression that tested positive for clomipramine and its main active metabolite. Quantitative relative recoveries (80–113%) were obtained by external calibration. The present work expands the applicability of the SBSDME to new analytes and new types of magnetic sorbents.

**Graphical abstract:**

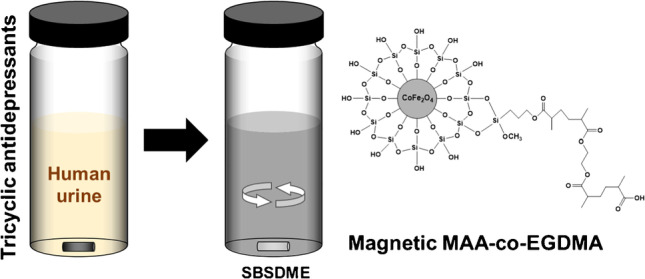

**Supplementary Information:**

The online version contains supplementary material available at 10.1007/s00604-021-05156-7.

## Introduction

Depression or major depressive disorder is one of the most common chronic or recurrent diseases which negatively affects human feelings, thoughts, and actions. Tricyclic antidepressants (TCAs) are a class of antidepressants traditionally prescribed for the treatment of this disease, but they have been widely replaced by newer drugs. However, either TCAs or some of their active metabolites (i.e., pharmacologically active and chemically stable metabolites resulting from the biotransformation of the parent drugs) are still prescribed not only for the treatment of depression, but also for other mental health issues [1]. Patients with major depression disorders often suffer overdose as a result of an uncontrolled self-consumption [2], and thus their detection and quantification in biological fluids are mandatory.

Traditionally, conventional liquid–liquid extraction (LLE) [3–5], solid-phase extraction (SPE) [6–8], and dilution/protein precipitation [9–11] are the most common pretreatment procedures applied to the analysis of TCAs in biological samples. However, these methods have many drawbacks such as the consumption of large amounts of sample and organic solvents, time-consuming procedures, and poor selectivity. Recently, microextraction techniques have alleviated these issues and different published methods can be found in the literature for TCAs determination [12, 13].

In general terms, those microextraction techniques based on magnetic (nano)materials, such as magnetic nanoparticles (MNPs) or composite materials made of the combination of MNPs and polymers, have taken on significance due to several reasons [14–16], especially for their easy retrieval by applying an external magnetic field, overcoming the time-consuming collection and handling of non-magnetic sorbents. In this context, the stir bar sorptive-dispersive microextraction (SBSDME) technique has attracted considerable attention [17]. In this technique, a magnetic (nano)material is added into a vial containing a magnetic neodymium stir bar, so that the magnetic sorbent is attracted by the magnet [18]. At high stirring rate, the magnetic sorbent is dispersed into the sample solution and, once the stirring is halted, the neodymium stir bar rapidly retrieves the sorbent containing the extracted analytes. Finally, when the extraction is completed, the magnetic sorbent–coated stir bar is easily removed from the sample solution and the analytes are desorbed in an adequate solvent (i.e., liquid desorption) or directly thermally desorbed into a gas chromatography (GC) system. The fundamentals of this approach, and an overview of the previously published SBSDME methods, have been recently compiled in a tutorial review [17].

On the other hand, it should be said that polymers, either purposely synthetized or commercially available, have been widely used as sorbents for solid-based microextraction techniques [19–21]. They can be tailored by using a high variety of monomers and, if it is the case, cross-linkers, thus achieving polymers with different properties. Furthermore, they can be tuned with different functional groups for specific purposes according to the extraction requirements [22]. In this respect, polymers made from methacrylic acid (MAA) and ethylene glycol dimethacrylate (EGDMA) as monomer and cross-linker, respectively, i.e., methacrylic acid-co-ethylene glycol dimethacrylate (MAA-co-EGDMA), have been widely used in many different approaches, such as monolith-based microextraction [23, 24], molecularly imprinted polymers-based SPE [25], solid-phase microextraction (SPME) [26, 27], or magnetic dispersive SPE [28]. These types of polymers present a hydrophobic chain structure functionalized with acidic groups, which confers them the capability to establish both hydrophobic and electrostatic interactions [24, 26] with basic compounds, such as TCAs. Moreover, if polymers are combined with MNPs, they confer the polymers the needed magnetism to easily handle them with magnetic fields. However, to the best of our knowledge, the combination of these polymers and magnetism has not been greatly exploited.

Thus, the aim of this work is the synthesis of a magnetic MAA-co-EGDMA polymer to be used in SBSDME for the extraction of TCAs. For this purpose, CoFe_2_O_4_ MNPs are chemically coated with SiO_2_ prior to the anchorage of vinyl moieties by reaction with 3-(trimethoxysilyl)propyl methacrylate (MPS), and the subsequent polymerization of the MAA-co-EGDMA copolymer on them (i.e., CoFe_2_O_4_@SiO_2_@MPS@MAA-co-EGDMA). Different polymerization mixtures, with different monomer:cross-linker molar ratios, were evaluated to obtain the most appropriate and mechanical resistant polymer. The chemistry of this interesting polymer was exploited for the extraction of the five TCAs most usually prescribed and their main active metabolites (Table S1, in Electronic Supplementary Material (ESM)) from urine samples. The main parameters affecting the extraction were carefully optimized through a response surface methodology (RSM), and those affecting the desorption were optimized by a univariate approach. Liquid chromatography–tandem mass spectrometry (LC–MS/MS) was used for analytes identification and quantification.

## Experimental

### Reagents

All reagents and solvents were obtained from major suppliers. Amitriptyline (AMT) hydrochloride ≥ 98%, nortriptyline (NORT) hydrochloride ≥ 98%, imipramine (IMP) hydrochloride ≥ 99%, desipramine (DIMP) hydrochloride ≥ 98%, trimipramine (TMP) maleate ≥ 98%, clomipramine (CMP) hydrochloride ≥ 98%, doxepin (DOX) hydrochloride ≥ 98%, methanolic solution containing 1000 µg mL^−1^ of N-desmethylclomipramine (NCMP) hydrochloride and methanolic solution containing 1000 µg mL^−1^ of cis/trans desmethyldoxepin (NDOX) from Sigma-Aldrich (St. Louis, USA, https://www.sigmaaldrich.com), and methanolic solution containing 1000 µg mL^−1^ of desmethyltrimipramine (NTMP) hydrochloride from LGC Standards (Luckenwalde, Germany, https://www.lgcstandards.com) were used as standards. Their chemical structures and relevant data are given in Table S1 (ESM).

For the synthesis of CoFe_2_O_4_ MNPs, cobalt(II) chloride hexahydrate (CoCl_2_·6H_2_O) and iron(III) chloride hexahydrate (FeCl_3_·6H_2_O) were purchased from Acros Organics (New Jersey, USA, https://www.acros.com), and sodium hydroxide (NaOH) reagent grade was purchased from Scharlau (Barcelona Spain, https://www.scharlau.com).

For the synthesis of the magnetic sorbent, tetraethylorthosilicate (TEOS) 98%, 3-(trimethoxysilyl) propyl methacrylate (MPS) 98%, and 2,2′-azobis(2-methylpropionitrile) (AIBN), methacrylic acid (MAA), and ethylene glycol dimethacrylate (EGDMA) from Sigma-Aldrich (Darmstadt, Germany) were used.

For the determination of the creatinine content in the urine samples, creatinine anhydrous ≥ 98% and picric acid moistened with water ≥ 98% were purchased from Sigma-Aldrich (St. Louis, USA).

HPLC grade absolute ethanol and HPLC grade acetonitrile from PanReac AppliChem (Damstadt, Germany, https://www.itwreagents.com), reagent grade ammonia and reagent grade hydrochloric acid from Scharlau (Barcelona, Spain), and ultrapure water (resistivity ≥ 18.2 MΩ · cm) obtained through of a Connect purification system from Adrona (Riga, Latvia, http://www.adrona.lv) have been used as solvents for the synthesis process.

Methanol (MeOH) grade LC–MS from VWR Chemicals (Fontenay-sous-Bois, France, https://es.vwr.com/store) has been used for the conditioning of the magnetic sorbent prior to extraction, and analytical grade sodium chloride (NaCl) (99.5%) from Acros Organics (Geel, Belgium) to adjust the ionic strength of the donor phase. Glacial acetic acid (AcOH) from Scharlau (Barcelona, Spain) has been used for the preparation of the solvent mixture for the liquid desorption.

The chromatographic mobile phase used was methanol grade LC–MS from VWR Chemicals (Fontenay-sous-Bois, France) and water grade LC–MS from Panreac (Barcelona, Spain), both with 0.1% formic acid LC–MS grade from VWR Chemicals.

Nitrogen, used as nebulizer and curtain gas in the MS/MS ion source, was obtained by a NiGen LC–MS 40.0 nitrogen generator from Claind S.r.l. (Lenno, Italy, https://www.claind.it/en/home). Extra pure nitrogen (> 99.999%) from Praxair (Madrid, Spain) was used as collision gas in the MS/MS collision cell.

### Samples

The analyte-free urine samples used for the development and validation of the method were obtained from different healthy volunteers who did not consume any type of antidepressants. In addition, a sample from a volunteer who did consume a prescribed drug containing 25 mg of CMP per tablet was analyzed. The amount ingested by this volunteer was one tablet at breakfast, one tablet at lunch, and two tablets at dinner. Each volunteer gave their written informed consent to participate in this study, which followed the ethical guidelines of the Declaration of Helsinki, and it was approved by the Ethical Committee of the University of Valencia (Spain). All urine samples were collected in sterile plastic containers and kept at 4 °C in the refrigerator until analysis.

### Apparatus and materials

An Agilent 1100 Series chromatography system comprising a degasser, a quaternary pump, an autosampler, and a thermostatic column oven coupled to an Agilent 6410B Triple Quad MS/MS was employed throughout the study.

A 10-position multiple stirring plate model MS-M-S10 from DLAB Scientific Europe S.A.S (Schiltigheim, France) and NdFeB magnets (54MGO, 10 mm length × 3 mm diameter) from Supermagnete (Gottmadingen, Germany) were used for SBSDME.

A ZX3 vortex mixer from VELP Scientifica (Usmate Velate, Italy) and an Ultrasons-HD ultrasonic bath from JP Selecta (Barcelona, Spain) were used in the synthesis stage.

A Jenway 6305 UV/Vis spectrophotometer from Cole-Parmer Ltd (Staffordshire, UK) was used for the determination of creatinine in urine samples.

A Basic 20 pH meter from Crison (Alella, Spain) was used for the pH adjustments.

All those instruments used for characterization of the sorbent material are listed in ESM.

### Synthesis of CoFe_2_O_4_@SiO_2_@MPS@MAA-co-EGDMA magnetic sorbent

**Fig. 1:**
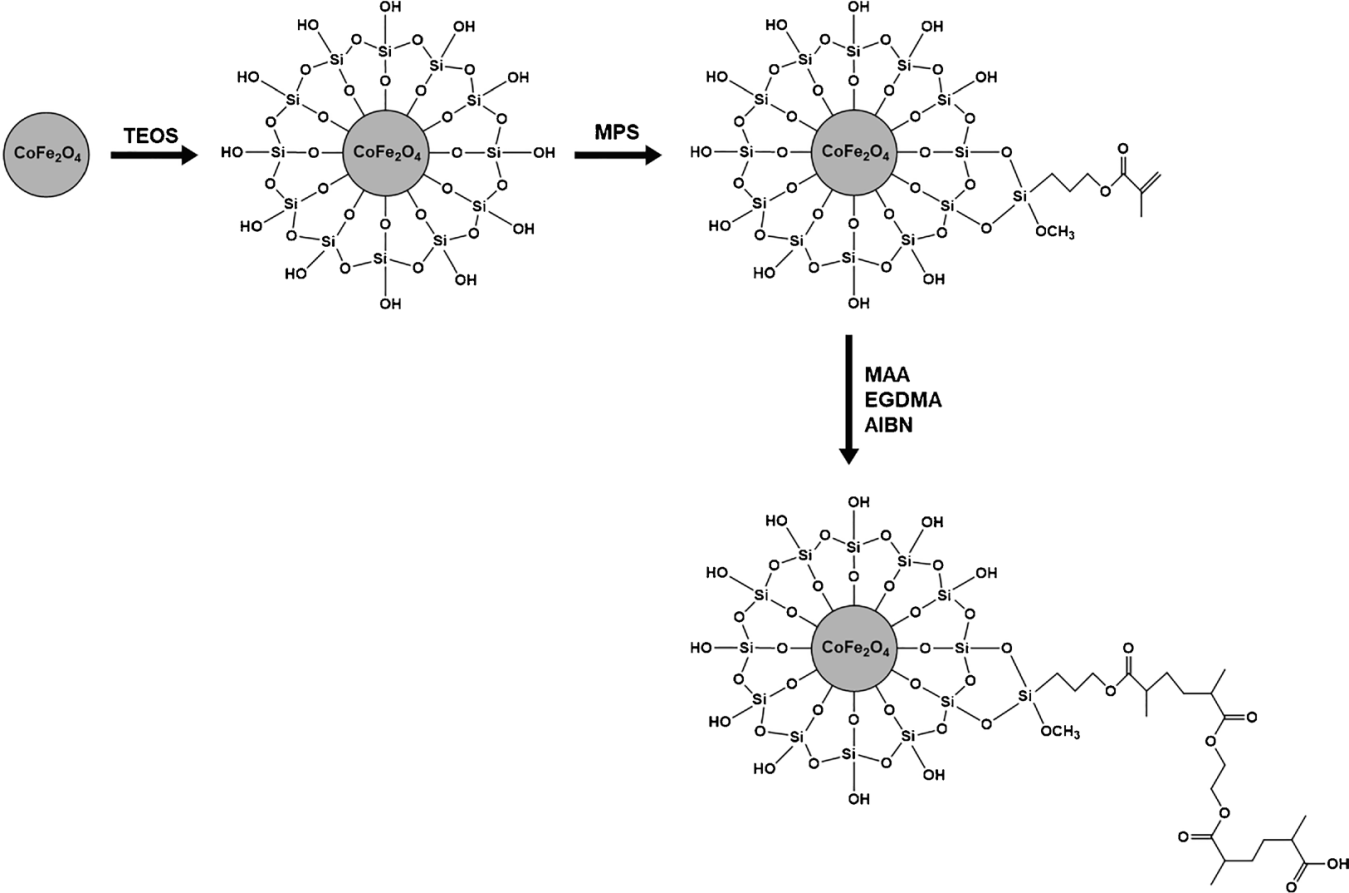
Synthesis of the CoFe_2_O_4_@SiO_2_@MPS@MAA-co-EGDMA sorbent

The synthesis of the CoFe_2_O_4_@SiO_2_@MPS@MAA-co-EGDMA sorbent consists of two global stages (Fig. 1): (1) the synthesis of the magnetic component, i.e., the CoFe_2_O_4_ MNPs, by chemical coprecipitation according to a modified protocol [29]; and (2) their chemical functionalization with the polymeric component (i.e., MAA-co-EGDMA). The adopted synthesis procedure consisted of a step-by-step process in order to ensure the chemical functionalization of the CoFe_2_O_4_ MNPs with the MAA-co-EGDMA polymer. In this sense, previous coatings with TEOS and then with MPS were needed to confer a SiO_2_ shell with reactive silanol groups and to provide functional vinyl moieties, respectively. Finally, the co-polymerization of MAA and EGDMA on these vinyl groups using AIBN as radical initiator was performed [30]. The EGDMA confers rigidity to the polymer and the MAA provides acidic functional groups available to interact with the basic analytes to the final sorbent.

Figure 1 schematizes the synthesis procedure of the magnetic sorbent, whereas the experimental details are described in ESM.

### Proposed method

#### External calibration and sample solutions

A 500-µg mL^−1^ multicomponent solution of the solid analytes (i.e., DOX, IMP, DIMP, AMT, TMP, NORT, and CMP) was prepared in MeOH. In parallel, from the 1000-µg mL^−1^ solutions of the rest of the analytes (i.e., NDOX, NTMP, and NCMP), individual solutions of 100 µg mL^−1^ in MeOH were prepared. From all the previous solutions, an intermediate multicomponent solution was prepared in water containing 5 µg mL^−1^ of all the analytes and, from this, another aqueous solution of 50 ng mL^−1^ was prepared. These solutions were stored at 4 °C protected from light exposure to avoid possible degradation. From the 50-ng mL^−1^ solution, working solutions (from 0.1 to 1 ng mL^−1^) were prepared in 6.8% w/v NaCl aqueous solution.

Prior to analysis, urine samples were conveniently diluted with deionized water, and NaCl was added to have 6.8% w/v in the measuring solution, thus ensuring that all analytes were at concentrations within the range of the study. In any case, at least 1:1 v/v dilution was required to avoid matrix effects (as discussed later).

#### Stir bar sorptive-dispersive microextraction procedure

**Fig. 2:**
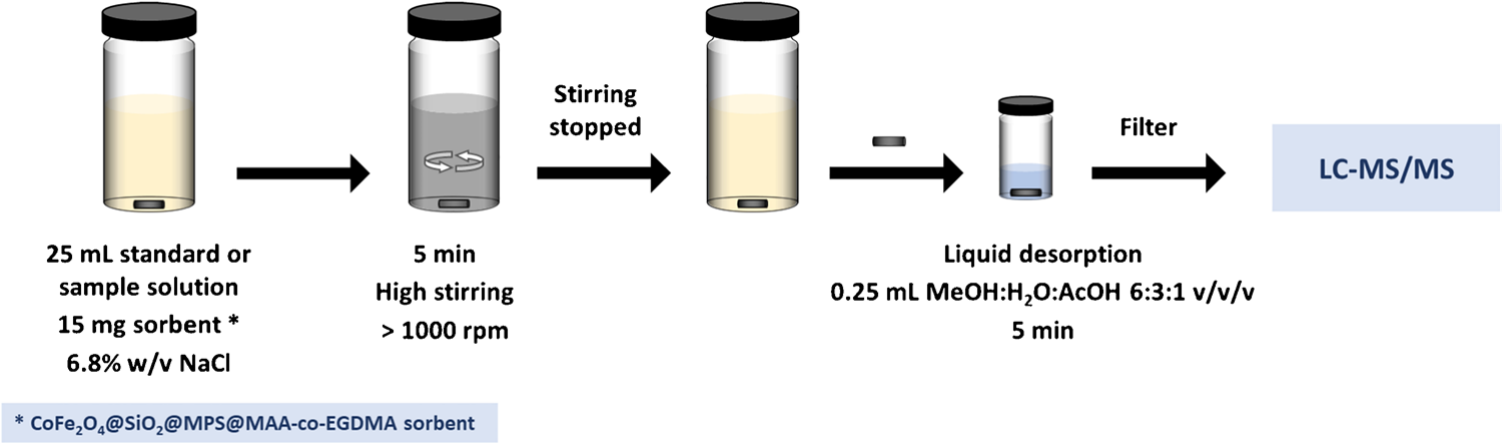
Schematic diagram of the experimental procedure

Prior to the extraction step, the CoFe_2_O_4_@SiO_2_@MPS@MAA-co-EGDMA sorbent was conveniently preconditioned. For this purpose, 15 mg of the sorbent was introduced into a clean and dry 40-mL vial and dispersed in 2 mL of MeOH with the help of a neodymium stir bar (10 mm length × 3 mm diameter) for 2 min. Once the stirring stopped, the magnetic material was strongly attracted to the stir bar. This stir bar coated with the material was removed from the solution with the help of plastic tweezers and placed into a 40-mL extraction vial. Then, 25 mL of each standard solution or diluted sample were introduced, respectively, to each vial containing the corresponding neodymium stir bar with the preconditioned sorbent. Then, all the vials were stirred by means of a 10-position multiple stirring plate for 5 min at a high-speed rate to allow the dispersion of the material into each solution at room temperature. After the extraction time elapsed, stirring was stopped and the magnetic material returned to the stir bar. Each sorbent-coated stir bar was then removed with plastic tweezers and placed in a 5-mL desorption vial containing 0.25 mL of MeOH:H_2_O:AcOH 6:3:1 v/v/v, as desorption solvent, to carry out the liquid desorption of the analytes. They were stirred for 5 min, and the extracts were filtered through a 0.22-µm nylon filter into injection vials. Finally, the extracts were analyzed by LC–MS/MS. Figure 2 shows a schematic diagram of the SBSDME procedure.

#### Liquid chromatography–tandem mass spectrometry analysis

The chromatographic separation was carried out in a Zorbax SB-C18 (50 mm length, 2.1 mm I.D., 1.8 µm) column. Five microliters of each extract were injected into the chromatographic system. The flow rate was 0.3 mL min^−1^, and the column temperature was kept constant at 35 °C. The mobile phase consisted of solvent A (H_2_O, 0.1% v/v formic acid) and solvent B (MeOH, 0.1% v/v formic acid), and the pump supplied the following gradient program: 0 to 1 min, linear gradient from 40 to 60% solvent B; held for 3.5 min; 4.5 to 4.6 min return to 40% solvent B and held for 5.4 min. The run time was 10 min.

The triple quadrupole MS detector operated in positive electrospray ionization mode (ESI^+^), at 3 kV, by multiple reaction monitoring (MRM). The flow rate and the temperature of the drying gas and the nebulizer pressure were 10 L min^−1^, 250 °C, and 25 psi, respectively. The m/z precursor → product ion transitions for quantification and for identification and the collision energies and fragmentor values for each analyte are shown in Table S2 (ESM).

Figure S1 (ESM) shows a chromatogram of a standard containing the analytes at 0.5 ng mL^−1^ obtained after applying the SBSDME-LC–MS/MS approach

## Results and discussion

### Selection of the monomer:cross-linker molar ratio

Results of the selection of the MAA:EGDMA molar ratio are discussed in ESM. Briefly, the 1:4 MAA:EGDMA molar ratio presented the highest extraction for all the target analytes, probably due to its high availability of acidic functional groups to interact with the basic analytes, and its high mechanical resistance. Thus, this sorbent was selected as sorbent for further analysis.

### Characterization of the CoFe_2_O_4_@SiO_2_@MPS@MAA-co-EGDMA magnetic sorbent

For the characterization of the selected CoFe_2_O_4_@SiO_2_@MPS@MAA-co-EGDMA magnetic sorbent, different techniques were used to study properties such as the magnetization (M_S_), the surface charge, the morphology, the adsorption properties, and the thermal stability. All these results are described in detail in ESM. In summary, the magnetization curve showed a satisfactory value of M_S_ (47.8 emu g^−1^) to carry out the SBSDME procedure. The point of zero charge (pH_pzc_), i.e., the pH at which the surface charge was zero, was determined to be ca. 3.3. The scanning electron microscopy (SEM) showed the expected spherical shape of the sorbent due to the successive coating of CoFe_2_O_4_ MNPs by SiO_2_, by MPS, and by the MAA-co-EGDMA polymer. The surface area was 60.7 ± 0.2 m^2^ g^−1^. Finally, the thermogravimetric analysis revealed that the sorbent was stable until 300 °C.

### Optimization of the extraction variables

The factors affecting the extraction procedure were optimized by using the RSM as a multivariate optimization method [31]. To this respect, a four-factor three-level Box-Benhken design was employed. Thus, the sorbent amount (*X*_1_), extraction time (*X*_2_), pH (*X*_3_), and ionic strength (*X*_4_) were considered as the potential factors affecting the extraction of the analytes, and they were optimized by performing 27 experiments (see ESM). The selected analytical responses were the peak areas of each analyte. StatGraphics Centurion XVI (Stat Point Inc. Herndon, VA, USA) software was employed for the statical analysis. The statistics of the description of the Box-Behnken design is included in ESM (see Table S3).

To this regard, sorbent amount (5–20 mg), extraction time (5–30 min), pH of the donor phase (2–8, adjusted by adding 0.035 g of H_2_NaPO_4_·H_2_O to 25 mL of aqueous solution and either o-phosphoric acid or ammonia), and ionic strength of the donor phase (0–10% NaCl) were evaluated. All the experiments were performed using 25 mL of aqueous standard solution at 5 ng mL^−1^. The desorption of the target analytes was carried out in 0.5 mL of MeOH:H_2_O:AcOH 6:3:1 v/v/v for 5 min.

Details of the optimization of the extraction variables are described in ESM. In short, according to RSM curves shown in Fig. S9 (ESM), there was no difference between 15 and 20 mg of CoFe_2_O_4_@SiO_2_@MPS@MAA-co-EGDMA, so 15 mg was selected as the optimum amount of sorbent. An extraction time of 5 min was enough to quantitatively extract the TCAs, so that it was selected for further experiments. Regarding the pH of the donor phase, optimum extraction values were observed between 5 and 7 pH values. At this range, an electrostatic interaction between the analytes and the sorbent is given due to the positive charge of the amine group of the TCAs (pKa 9.2–10.4) and the negatively charged surface of the sorbent (pH_pzc_ 3.3). Therefore, it would not be mandatory to be adjusted since the pH of the diluted urine is nearby 6. Finally, the optimum ionic strength was achieved at 6.8% w/v NaCl.

### Optimization of the desorption variables

The desorption conditions were also evaluated, in triplicate, using a univariate approach under the extraction conditions previously optimized. In this regard, the desorption solvent, desorption time, and desorption volume were selected as the variables affecting the desorption of the target analytes. In this case, the selected analytical response was the enrichment factor (EF), defined as EF = *A*_ext_/*A*_0_, where *A*_ext_ is the peak area of each target analyte after the extraction procedure (i.e., in the final extract), and *A*_0_ is the initial peak area of this analyte (i.e., in the donor solution before the extraction).

Details of the optimization of the desorption variables are discussed in ESM. Briefly, the best EF values were achieved when 0.25 mL of MeOH:H_2_O:AcOH 6:3:1 v/v/v was used for 5 min during the desorption process (see Figs. S10–S12 (ESM)). This is probably due to the low pH level (ca. 2.2) caused by the AcOH, which leads to an electrostatic repulsion between the positively charged surface of the sorbent (pH_pzc_ of 3.3) and the positively charged amine groups of the TCAs (pKa 9.2–10.4).

### Extraction efficiency of the CoFe_2_O_4_@SiO_2_@MPS@MAA-co-EGDMA sorbent

In order to demonstrate the extraction efficiency of the MAA-co-EGDMA polymer within the synthesized magnetic sorbent, bare CoFe_2_O_4_ MNPs, MAA-co-EGDMA polymer, and CoFe_2_O_4_@SiO_2_@MPS@MAA-co-EGDMA magnetic polymer were tested as sorbent materials under the optimized extraction and desorption conditions. Since the polymer is not magnetic by itself, its retrieval after extraction was performed by centrifugation for 5 min at 6000 rpm. The extraction efficiency was estimated according to Eq. (1) described in the ESM, and these results are shown in Fig. S13 (ESM). As can be seen, the % extraction of bare CoFe_2_O_4_ MNPs is minimal when compared to that of the MAA-co-EGDMA polymer. Moreover, the presence of the coated CoFe_2_O_4_ MNPs in the final magnetic sorbent did not affect the extraction efficiency of the MAA-co-EGDMA polymer.

### Inter-batch repeatability of the CoFe_2_O_4_@SiO_2_@MPS@MAA-co-EGDMA sorbent

The repeatability between different batches of the CoFe_2_O_4_@SiO_2_@MPS@MAA-co-EGDMA sorbent was also evaluated. For this purpose, three replicates of an aqueous standard solution containing 250 ng L^−1^ of the analytes were extracted, under the optimized conditions, with three different batches of the sorbent synthesized as described in “Synthesis of CoFe_2_O_4_@SiO_2_@MPS@MAA-co-EGDMA magnetic sorbent.” As can be seen in Fig. S14 (ESM), similar results were obtained with the three batches, so it can be concluded that the synthesis of the material is repeatable and provides batches with very similar extraction capability.

### Study of the matrix effects

In order to study the matrix effect of the urine samples during the analytical process, the slopes of the calibration plots obtained in water (external calibration) and those obtained using a pool of three free-analyte urine samples (matrix-matched calibration), both subjected to the optimized SBSDME procedure, were compared for a 5% significance level by applying the Student’s *t*-test [32]. The slopes obtained for both calibration methods are shown in Table S4 (ESM). Given that the obtained results suggested negative matrix effects, a dilution of the urine sample was tested in order to avoid matrix effects. In this sense, different dilutions were studied to find out at what dilution the matrix effect was negligible. As can be seen in Fig. S15 (ESM), the matrix effect was negligible when a 1:1 v/v dilution or higher was used. Therefore, an external calibration can be used as long as the urine sample was diluted at least at 1:1 v/v.

### Analytical figures of merit

Method validation was performed evaluating different parameters, such as linearity, instrumental and method limits of detection (ILOD and MLOD, respectively) and quantification (ILOQ and MLOQ, respectively), EF, and repeatability (expressed as relative standard deviation (RSD)), under the optimized conditions defined above. These parameters are shown in Table 1.

**Table 1:** Main quality parameters of the proposed SBSDME-LC–MS/MS method

TCA	*R*^2a^	EF^b^		ILOD^c^ (ng L^−1^)	ILOQ^c^ (ng L^−1^)	MLOD^d^ (ng L^−1^)	MLOQ^d^ (ng L^−1^)	Repeatability (% RSD) ^e^					
								Intra-day			Inter-day		
								50 ng L^−1^	250 ng L^−1^	1000 ng L^−1^	50 ng L^−1^	250 ng L^−1^	1000 ng L^−1^
DOX	0.9990	22	7.0		23.1	14.0	46.1	6.9	2.7	5.1	10.6	13.3	5.5
NDOX	0.9993	13	5.3		17.6	10.6	35.1	7.3	4.3	6.5	0.7	9.6	10.4
IMP	0.9994	16	2.3		7.7	4.7	15.4	9.3	2.1	7.0	12.8	12.5	4.7
DIMP	0.9992	16	1.4		4.7	2.9	9.4	4.0	1.9	5.0	7.0	14.7	2.0
AMT	0.9998	19	6.2		20.4	12.4	40.8	5.9	1.3	4.7	12.4	6.0	2.1
TMP	0.9994	17	2.7		8.9	5.4	17.7	4.2	1.0	5.3	14.7	14.2	6.1
NORT	0.9996	18	2.2		7.3	4.4	14.5	2.2	2.0	5.3	12.9	9.1	1.4
NTMP	0.9997	19	3.0		9.9	6.0	19.7	1.4	2.4	5.2	3.2	15.4	4.4
CMP	0.9998	21	2.5		8.3	5.0	16.5	9.8	2.7	5.3	9.5	14.1	12.5
NCMP	0.997	21	2.0		6.6	4.0	13.2	3.3	3.4	3.0	6.8	8.5	7.6

^a^*R*^2^ coefficient of determination. Working range: 100–1000 ng L^−1^; number of calibration points: 6

^b^*EF* enrichment factor, obtained with an aqueous standard solution containing 250 ng L^− 1^ of the target analytes

^c^*ILOD* instrumental limit of detect ion; *ILOQ* instrumental limit of quantification; calculated as 3 times and 10 times, respectively, the signal-to-noise ratio

^d^*MLOD* method limit of detection; *MLOQ* method limit of quantification; considering a 1:1 v/v dilution of the urine sample

^e^*RSD* relative standard deviation (*n* = 5)

A high level of linearity was accomplished, reaching at least up to 50 ng mL^−1^, with coefficients of determination (*R*^2^) > 0.997.

The ILODs and ILOQs were calculated as 3 times and 10 times, respectively, the signal-to-noise ratio. They ranged from 1.4 to 7.0 ng L^−1^, and from 4.7 to 23.1 ng L^−1^, respectively. On the other hand, MLODs and MLOQs were obtained considering a 1:1 v/v dilution of the urine sample. Thus, these values ranged from 2.9 to 14.0 ng L^−1^, and from 9.4 to 46.1 ng L^−1^, respectively.

The EF values were estimated as mentioned above and taking into account a 1:1 v/v dilution of the urine sample. Hence, the net EFs values ranged from 13 to 22.

Finally, the precision of the proposed SBSDME method was calculated from the analysis of five independent replicate aqueous standard solutions containing the target analytes at three different concentration levels (i.e., 50, 250, and 1000 ng L^−1^) in the same day (intra-day repeatability) and in five consecutive days (inter-day repeatability). RSD values, ranged from 0.7 to 15.4%, showed the good precision of the proposed method.

Compared to other reported nanomaterial-based methods for the determination of TCAs in human urine (see Table 2), the proposed SBSDME approach presents different advantages. The analytical performance in terms of MLODs, even when thermal desorption was used, in addition to relative recoveries and precision, is comparable or better than previously reported methods. Furthermore, the highest number of TCAs were determined by the proposed method, including their main metabolites in urine. The overall extraction plus desorption time is similar to other dynamic extraction techniques whereas, as expected, is shorter when compared to static ones. Moreover, no additional equipment except a stirring plate and a bar-shaped magnet is needed to perform the extraction and rapid retrieval of the material, thus avoiding centrifugation steps making it fast and easy to handle.

**Table 2:** An overview on reported nanomaterial-based methods for the determination of tricyclic antidepressants in human urine

Analytes^a^	Microextraction technique^b^	Sorbent material^c^	Time^d^	Instrumental technique^e^	Analytical performance^f^	Ref
DOX, IMP, CMP, and other three compounds	PMME	Poly(MAA-co-EGDMA) in combination with OPA-modified Zr-coated CEC	9 min	CE-UV	EF: n.a MLOD: 3.7–8.0 ng mL^−1^ RR: 84–107% RSD: 0.6–9.4%	[33]
IMP, DIMP, CMP	SPME	G, CTAB and PANI nanocomposite	60 min	TD-GC-FID	EF: n.a MLOD: 0.10–0.35 ng mL^−1^ RR: 94–99% RSD: 4.8–10.4%	[34]
AMT, IMP, and another compound	MEPS	PDA-Ag-PPy nanocomposite	15 min	GC–MS	EF: n.a MLOD: 0.03–0.05 ng mL^−1^ RR: 88–104% RSD: 5–10%	[35]
DOX, AMT and NORT	DSPE-CAE	Fe_3_O_4_@oleic acid	19 min	LC-UV	EF: n.a : 0.5–1.4 ng mL^−1^ RR: 87–105% RSD: 2.5–3.2%	[36]
AMT, DIMP, TMP, and another compound	Micro-SPE	Poly(GMA-co-EDMA-MWCNTs)	5 min	LC-UV	EF: 24–36 MLOD: 8.6–15.2 ng mL^−1^ RR: 72–108% RSD: 3–14%	[37]
AMT, IMP	MSPE	Fe_3_O_4_@TMU-10	32 min	LC-UV	EF: 48–50 MLOD: 2–5 ng mL^−1^ RR: 96–99% RSD: 3.5–4.7%	[38]
AMT and NORT	MSPE	Fe_3_O_4_@SiO_2_@N_3_	5 min	LC-UV	EF: n.a MLOD: 0.03–0.05 ng mL^−1^ RR: 95–97% RSD: 1.1–3.7%	[39]
DOX, AMT, NORT	SPME	POMo_368_/PANI composite	40 min	LC-UV	EF: n.a : < 0.0002 ng mL^−1^ RR: 92–98% RSD: 4.1–5.9%	[40]
DOX, NDOX, IMP, DIMP, AMT, TMP, NORT, NTMP, CMP, and NCMP	SBSDME	CoFe_2_O_4_@SiO_2_@MPS@MAA-co-EGDMA	10 min	LC–MS/MS	EF: 13–22 MLOD: 0.0029–0.014 ng mL^−1^ RR: 80–113% RSD: 0.7–15.4%	This work

^a^*AMT* amitriptyline; *CMP* clomipramine; *DIMP* desipramine; *DOX* doxepin; *IMP* imipramine; *NCMP* N-desmethylclomipramine; *NDOX* N-desmethyldoxepin; *NORT* nortriptyline; *NTMP* N-desmethyltrimipramine; *TMP* trimipramine

^b^*CTAB* cetyl trimethylammonium bromide; *EDMA* ethylene dimethacrylate; *EGDMA* ethylene glycol dimethacrylate; *G* graphene; *GMA* glycidyl methacrylate; *MAA* methacrylic acid; *MPS* 3-(trimethoxysilyl)propyl methacrylate; *MWCNTs* multi-walled carbon nanotubes; *OPA* octadecyl phosphonic acid; *PANI* polyaniline; *PDA* polydopamine; *POMo*_*368*_ polyoxomolibdate_368_; *PPy* polypyrrole

^c^*DSPE-CAE* dispersive solid-phase extraction with coacervative microextraction; *MEPS* microextraction in package syringe; *MSPE* magnetic solid-phase extraction; *PMME* polymer monolith microextraction; *SBSDME* stir bar sorptive-dispersive microextraction; *SPE* solid-phase extraction; *SPME* solid-phase microextraction

^d^Time: extraction + desorption time

^e^*CE* capillary electrophoresis; *FID* flame ionization detector; *GC* gas chromatography; *LC* liquid chromatography; *MS* mass spectrometry; *MS/MS* tandem mass spectrometry; *TD* thermal desorption; *UV* ultraviolet spectrometry

^f^*EF* enrichment factor; *MLOD* method limit of detection; *n.a.* not available; *RR* relative recovery; *RSD* relative standard deviation

On the other hand, the main drawback of the proposed method is the step-by-step synthesis procedure of the sorbent material, not being able to synthesize it in a single step. However, the proposed synthesis procedure allows to obtain large amounts of sorbent (> 1.5 g), and considering the small amount of material that was used in each extraction (i.e., 15 mg), it allows to carry out many extractions in just a single synthesis. In addition, as the polymer is chemically coating MNPs, rather than having the MNPs physically embedded into it, the contact area between analytes and sorbent is increased due to nanometric size and, moreover, leaching problems are minimized.

### Application to the analysis of real human urine samples

The proposed SBSDME method was applied to the analysis of the five target TCAs and their main metabolites in three real human urine samples in order to evaluate its accuracy and analytical utility. In this sense, the proposed method was applied to one urine sample from a volunteer (Volunteer A) consuming a prescribed drug, which contained 25 mg of CMP per tablet, three times per day, and two urine samples from two healthy volunteers (Volunteers B and C) who did not take any TCA. As expected, only the urine belonging to Volunteer A contained CMP and its main metabolite (NCMP). Thus, 0.28 ± 0.03 and 1.64 ± 0.10 µg mg^−1^ of creatinine for CMP and NCMP, respectively, were found.

Regarding the accuracy of the proposed method (Table 3), these three samples were spiked at two concentration levels (i.e., 200 and 400 ng L^−1^) of the target analytes, showing good relative recovery values (80–113%). Therefore, the external calibration approach works in a reliable way.

**Table 3:** Relative recovery values obtained by applying the proposed SBSDME-LC–MS/MS method

TCA	Added (ng mL^−1^)	Volunteer A		Volunteer B		Volunteer C	
		Found (ng mL^−1^)	Relative recovery (%)	Found (ng mL^−1^)	Relative recovery (%)	Found (ng mL^−1^)	Relative recovery (%)
DOX	0.00	n.d.^a^	-	n.d.^a^	-	n.d.^a^	-
	0.23	0.211 ± 0.017	91 ± 7	0.209 ± 0.008	90 ± 4	0.192 ± 0016	83 ± 7
	0.46	0.464 ± 0.012	100 ± 3	0.429 ± 0.013	92 ± 3	0.463 ± 0.005	100 ± 1
NDOX	0.00	n.d.^a^	-	n.d.^a^	-	n.d.^a^	-
	0.20	0.169 ± 0.005	84 ± 3	0.170 ± 0.019	85 ± 9	0.161 ± 0.005	81 ± 3
	0.40	0.342 ± 0.015	85 ± 4	0.35 ± 0.03	87 ± 7	0.366 ± 0.012	92 ± 3
IMP	0.00	n.d.^a^	-	n.d.^a^	-	n.d.^a^	-
	0.21	0.204 ± 0.009	103 ± 12	0.172 ± 0.001	81 ± 1	0.174 ± 0.007	82 ± 4
	0.42	0.41 ± 0.03	97 ± 7	0.343 ± 0.003	81 ± 1	0.340 ± 0.007	80 ± 2
DIMP	0.00	n.d.^a^	-	n.d.^a^	-	n.d.^a^	-
	0.20	0.210 ± 0.005	108 ± 9	0.180 ± 0.010	88 ± 5	0.168 ± 0.015	82 ± 7
	0.41	0.41 ± 0.03	101 ± 6	0.340 ± 0.017	83 ± 4	0.371 ± 0.005	91 ± 1
AMT	0.00	n.d.^a^	-	n.d.^a^	-	n.d.^a^	-
	0.21	0.216 ± 0.004	106 ± 2	0.167 ± 0.008	81 ± 4	0.165 ± 0.006	81 ± 3
	0.41	0.420 ± 0.005	102 ± 1	0.329 ± 0.008	80 ± 2	0.348 ± 0.001	85 ± 1
TMP	0.00	n.d.^a^	-	n.d.^a^	-	n.d.^a^	-
	0.21	0.23 ± 0.02	107 ± 11	0.192 ± 0.009	91 ± 4	0.176 ± 0.011	83 ± 5
	0.42	0.437 ± 0.018	103 ± 4	0.373 ± 0.013	88 ± 3	0.392 ± 0.002	92 ± 1
NORT	0.00	n.d.^a^	-	n.d.^a^	-	n.d.^a^	-
	0.20	0.200 ± 0.018	92 ± 4	0.180 ± 0.011	92 ± 6	0.163 ± 0.011	83 ± 5
	0.39	0.394 ± 0.010	100 ±	0.338 ± 0.013	86 ± 3	0.360 ± 0.003	92 ± 1
NTMP	0.00	n.d.^a^	-	n.d.^a^	-	n.d.^a^	-
	0.20	0.188 ± 0.015	94 ± 8	0.161 ± 0.009	80 ± 5	0.160 ± 0.004	83 ± 5
	0.40	0.38 ± 0.02	95 ± 5	0.320 ± 0.023	80 ± 6	0.328 ± 0.001	92 ± 1
CMP	0.00	0.11 ± 0.01	-	n.d.^a^	-	n.d.^a^	-
	0.21	0.32 ± 0.05	113 ± 6	0.185 ± 0.013	89 ± 7	0.169 ± 0.002	81 ± 1
	0.42	0.538 ± 0.009	101 ± 2	0.353 ± 0.014	84 ± 3	0.404 ± 0.008	96 ± 2
NCMP	0.00	0.66 ± 0.04	-	n.d.^a^	-	n.d.^a^	-
	0.20	0.87 ± 0.03	102 ± 13	0.179 ± 0.020	89 ± 10	0.167 ± 0.013	83 ± 7
	0.40	1.071 ± 0.016	102 ± 4	0.336 ± 0.018	84 ± 5	0.382 ± 0.001	96 ± 1

^a^*n.d.* not detected

## Conclusions

In the present work, an optimized SBSDME method that contributes to the development of sensitive methods for the determination of five TCAs and their main metabolites in urine samples has been presented. The use of CoFe_2_O_4_@SiO_2_@MPS@MAA-co-EGDMA as sorbent phase provides good extraction of the proposed TCAs, through both hydrophobic and electrostatic interactions. This is the first time that this interesting polymer has been used in the SBSDME approach and also for the determination of these compounds. Moreover, due to the features of this polymer, the proposed magnetic sorbent may be used in new applications to extract many other families of basic compounds containing, for instance, amino functional groups. The proposed SBSDME method has been validated in terms of linearity, enrichment factor, LODs and LOQs, repeatability, and accuracy, obtaining good results.

This work expands the analytical potential of SBSDME to other analytes and to the use of new sorbent phases in this technique, demonstrating its versatility regardless the chemical nature of the analytes.

## Supplementary Information

Below is the link to the electronic supplementary material.

Supplementary file1 (PDF 2335 KB)
