# Supplementary material for: Stir bar sorptive-dispersive microextraction by a poly(methacrylic acid-co-ethylene glycol dimethacrylate)-based magnetic sorbent for the determination of tricyclic antidepressants and their main active metabolites in human urine
Source: Mikrochim Acta. 2022 Jan 8;189(2):52. doi: 10.1007/s00604-021-05156-7 (PMC8742809; doi:10.1007/s00604-021-05156-7)
Supplement: Supplementary file 1 — Supplementary file1 (PDF 2335 KB) [file 604_2021_5156_MOESM1_ESM.pdf]

Electronic Supplementary Material (ESM)

Stir bar sorptive dispersive microextraction by a poly(methacrylic acid-co-ethylene glycol dimethacrylate)-based magnetic sorbent for the determination of tricyclic antidepressants and their main active metabolites in human urine

Víctor Vállez-Gomis, Sara Exojo-Trujillo, Juan L. Benedé, Alberto Chisvert, Amparo Salvador

GICAPC Research group, Department of Analytical Chemistry,
University of Valencia, 46100 Burjassot, Valencia, Spain

* Corresponding author:

E-mail address: alberto.chisvert@uv.es (A. Chisvert)

TABLE OF CONTENTS

Chemical structure and relevant data of the target compounds 2

Synthesis of the $CoFe_2O_4$@SiO_2@MPS@MAA-co-EGDMA sorbent 3

Liquid chromatographic-tandem mass spectrometry analysis 4

Selection of the monomer:cross-linker molar ratio 6

Characterization of the $CoFe_2O_4$@SiO_2@MPS@MAA-co-EGDMA sorbent 7

Instruments 7

Magnetization curve 7

Zeta potential – point of zero charge 8

Morphology 8

Adsorption properties 9

Thermogravimetric analysis 10

Box-Behnken design 11

Optimization of the extraction variables 13

Optimization of the desorption variables 16

Desorption solvent 16

Desorption time 17

Desorption volume 18

Extraction efficiency of the $CoFe_2O_4$@SiO_2@MPS@MAA-co-EGDMA sorbent 19

Inter-batch repeatability of the $CoFe_2O_4$@SiO_2@MPS@MAA-co-EGDMA sorbent 19

Study of matrix effects 20

Chemical structure and relevant data of the target compounds

Table S1. Chemical structure and relevant data of the target compounds.

TCA	Chemical structure	CAS	$logP_{ow}$ [a]	pK_a [b]
Doxepin (DOX)	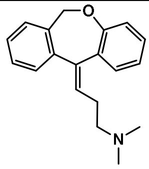	1668-19-5	3.84	9.4
Nordoxepin (NDOX)	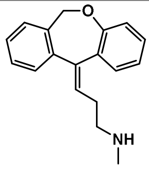	1225-56-5	3.40	10.3
Imipramine (IMP)	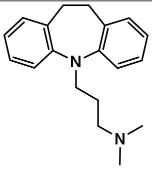	50-49-7	4.36	9.5
Desipramine (DIMP)	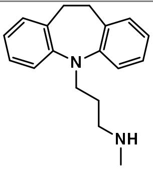	50-47-5	3.97	10.4
Amitriptyline (AMT)	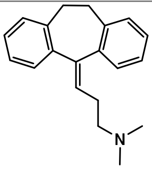	50-48-6	4.41	9.2
Trimipramine (TMP)	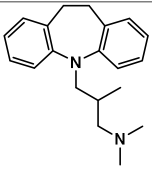	739-71-9	4.71	9.4
Nortriptyline (NORT)	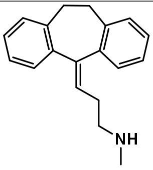	72-69-5	3.97	10.0
Nortrimipramine (NTMP)	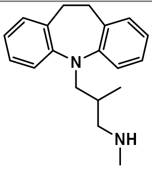	2293-21-2	4.33	10.4
Clomipramine (CMP)	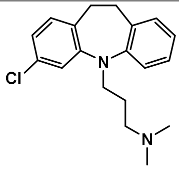	303-49-1	4.94	9.5
Norclomipramine (NCMP)	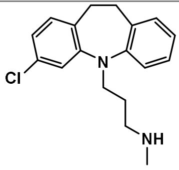	303-48-0	4.56	10.4

[a] P_{ow}: Octanol-water partition coefficient

[b] K_a: Acid dissociation constant

Synthesis of the $CoFe_2O_4$@SiO_2@MPS@MAA-co-EGDMA sorbent

First of all, the cobalt ferrite nanoparticles were synthesized by dissolving 10.80 g of $FeCl_3 \cdot 6H_2O$ and 4.76 g of $CoCl_2 \cdot 6H_2O$ in 200 mL of water. This solution was homogenized by magnetic stirring and heated to 80 ºC. Then, 100 mL of a 3 M NaOH solution were added dropwise, maintaining stirring for 1 h more. Subsequently, it was left to cool down up to room temperature and the solid was magnetically decanted. Then, it was washed with water until neutral pH, and afterwards with ethanol three times. Finally, the $CoFe_2O_4$ MNPs were dried overnight at 80 ºC and pulverized until obtaining a fine black powder.

For the synthesis of the $CoFe_2O_4$@SiO_2, 3.68 g of $CoFe_2O_4$ MNPs were weighed in a 500 mL beaker, and a mixture of 300 mL of water and 60 mL of ethanol was added. Then, the $CoFe_2O_4$ MNPs were dispersed for 30 min by means of ultrasounds. Next, 75 mL of 28% w/w aqueous ammonia solution and 18 mL of TEOS were added, and the reaction mixture was magnetically stirred for 12 h at room temperature. Finally, the precipitate was washed several times with water and ethanol, it was dried overnight in an oven at 80 ºC and it was pulverized until a fine powder was obtained.

For the functionalization of the silica surface with vinyl groups by means of MPS, 4.34 g of $CoFe_2O_4$@SiO_2 were introduced into a round bottom flask and dispersed in 200 mL of water and 20 mL of 0.01 M HCl by magnetic stirring. This mixture was then purged with dry nitrogen for 15 min. Next, 2.15 mL of MPS, previously purged with nitrogen too, were added and the mixture was magnetically stirred for 24 h at room temperature. Finally, the precipitate was washed several times with water and ethanol, it was dried overnight in an oven at 80 ºC and it was pulverized until a fine powder was obtained.

For the co-polymerization of MAA and EGDMA, 0.125 g of AIBN and 1 g of $CoFe_2O_4$@SiO_2@MPS were weighed and placed in a round bottom flask. Then, 250 mL of acetonitrile, 2.27 mL of EGDMA and 0.51 mL of MAA were sequentially added, and then the mixture was dispersed in an ultrasounds bath for 30 min and purged with dry nitrogen for 15 min. Finally, it was sealed and stirred for 24 h at 60 ºC. After that time, the solid formed was washed with acetonitrile and ethanol several times, dried overnight at 80 ºC and the obtained dark gray solid was pulverized until a fine powder was obtained.

Liquid chromatographic-tandem mass spectrometry analysis

Table S2. Tandem mass spectrometry parameters for the target analytes.

TCA	Precursor ion (m/z)	Product ion (m/z) [a]	Collision energy (V)	Fragmentor (V)
DOX	280.17	**107.0**	22	132
		77.1	62	
NDOX	266.16	**107.0**	18	132
		77.1	62	
IMP	281.20	**86.1**	14	114
		58.1	46	
DIMP	267.19	**72.1**	10	96
		193.1	38	
AMT	278.19	**91.0**	26	132
		105.0	22	
TMP	295.22	**100.1**	14	114
		58.1	46	
NORT	264.18	91.0	22	114
		**233.1**	10	
NTMP	281.20	**86.1**	14	114
		193.1	42	
CMP	315.16	**86.1**	14	132
		58.1	46	
NCMP	301.15	**72.1**	14	114
		227.0	38	

[a] The m/z values used as quantifiers are marked in bold

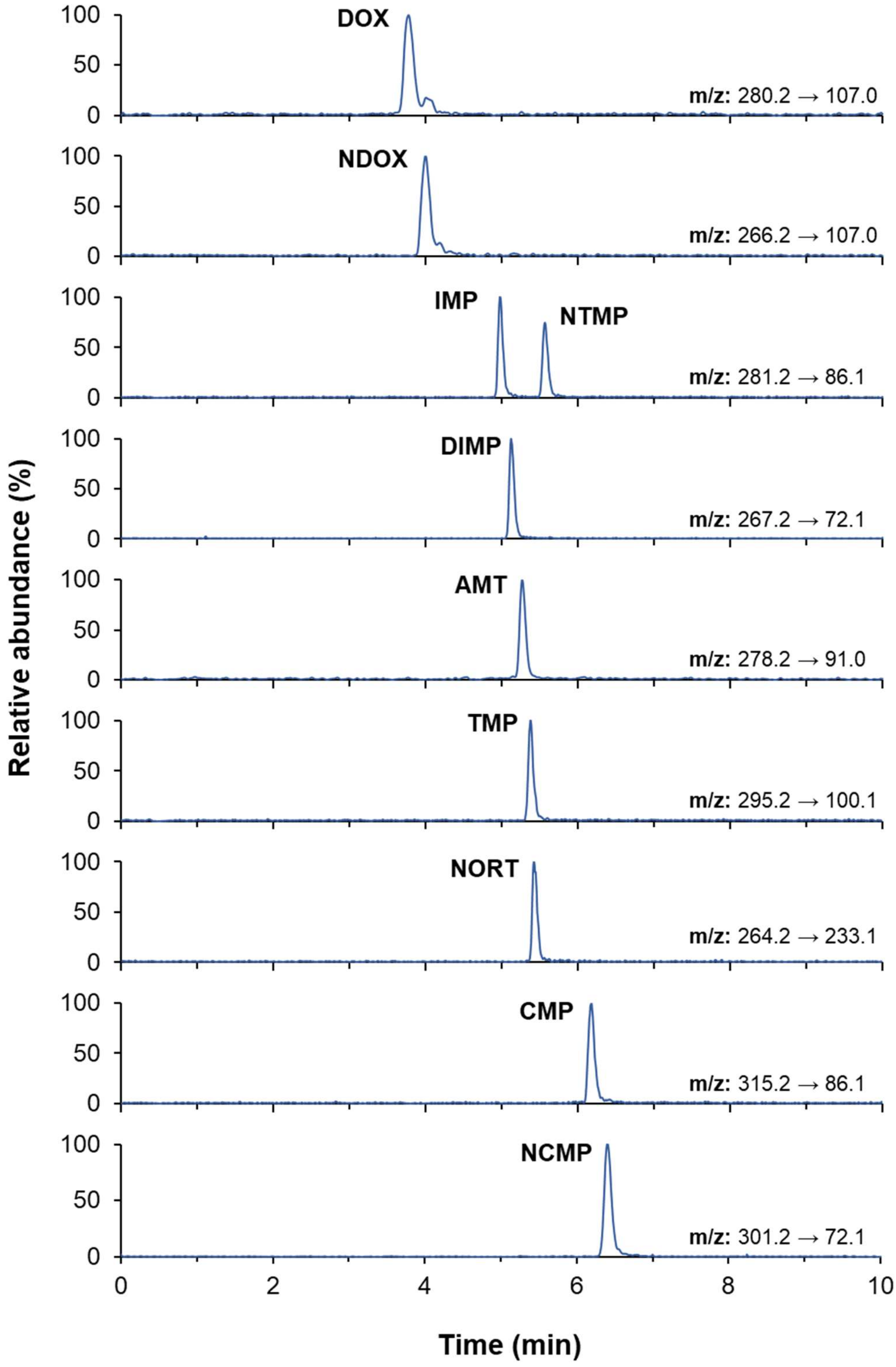

Fig. S1. Chromatogram of a standard solution containing the target analytes at 0.5 ng mL^{-1} obtained by the proposed method.

Selection of the monomer:cross-linker molar ratio

Before the optimization of the SBSDME procedure, different monomer:cross-linker (i.e., MAA and EGDMA, respectively) molar ratios were evaluated in order to obtain the most appropriate and mechanical resistant polymer. In this sense, the studied monomer:cross-linker molar ratios were 1:2, 1:4 and 1:6. All the experiments were performed, in triplicate, using 25 mL of aqueous standard solution at 5 ng mL^{-1} of the target analytes. The selected analytical response was the % extraction, which is defined as follows:

$$\% \text{ Extraction} = 100 - \left(\frac{A_{DP}}{A_0} \cdot 100\right) \quad (1)$$

where A_{DP} is the peak area of each target analyte in the donor phase after the extraction procedure, and A_0 is the initial peak area of this target analyte (i.e., in the donor solution before the extraction).

As can be seen in Fig. S2, the 1:4 monomer:cross-linker molar ratio presented the highest % extraction for all the target analytes. This is probably due, on the one hand, to the higher availability of acidic functional groups than in 1:6 ratio and thus interacting more with the basic analytes, and on the other hand, to the higher mechanical resistance than 1:2 ratio. Thus, the 1:4 monomer:cross-linker molar ratio was selected as sorbent for further analysis.

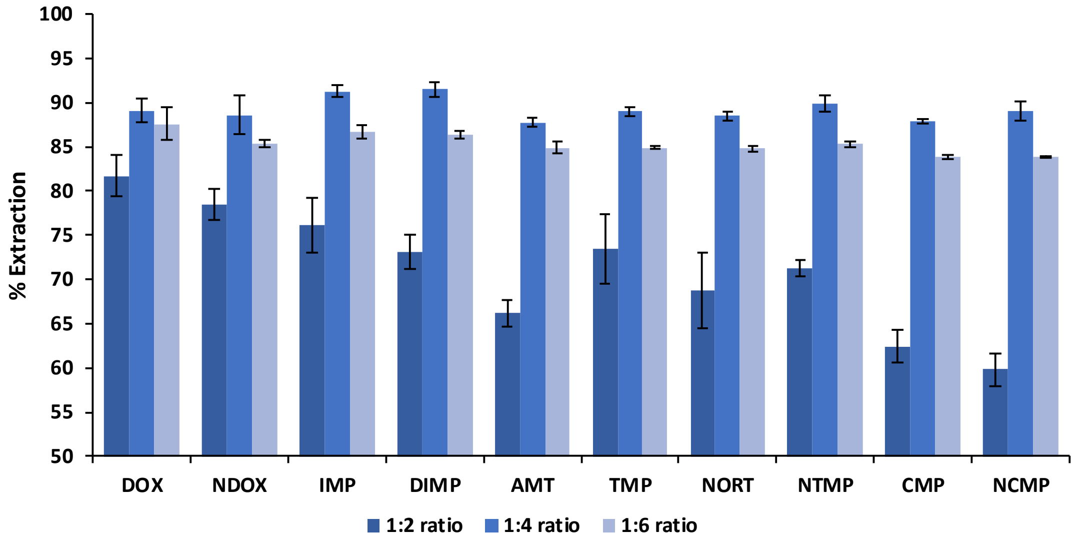

Fig. S2. Study of the MAA:EGDMA ratio. Extraction conditions: sorbent amount, 5 mg; extraction time, 5 min; pH, not adjusted; ionic strength, not adjusted.

Characterization of the $CoFe_2O_4$@SiO_2@MPS@MAA-co-EGDMA sorbent

Instruments

A Quantum Design (CA, USA) MPMS-XL-5 superconducting quantum interference device (SQUID) magnetometer was used to measure the magnetic properties of the sorbent.

Zeta potential measurements in order to determine the point of zero charge of the magnetic sorbent were performed using a Malvern Zetasizer ZS instrument.

A HITACHI S4800 scanning electron microscopy (SEM) operating at 10 kV equipped with an RX Bruker nackscattered electron detector was used to observe the morphology.

Nitrogen adsorption-desorption isotherms were measured on an ASAP 2010 analyzer from Micromeritics (GA, USA) in order to determine the surface area.

Thermogravimetric analysis (TGA) was accomplished using a Perkin-Elmer TGA-7 thermobalance by heating from room temperature to 1000 ºC under O_2 atmosphere.

Magnetization curve

Regarding the magnetization curve (Fig. S3), it showed a saturation magnetization (M_S) about 47.8 emu g^{-1}, which was appropriate to be used in SBSDME. A residual magnetism (retentivity) of 24 emu g^{-1}, and a coercivity about 1.5 kOe to demagnetize the material after its magnetization were obtained. According to these results, the $CoFe_2O_4$@SiO_2@MPS@MAA-co-EGDMA sorbent is a soft ferromagnetic material.

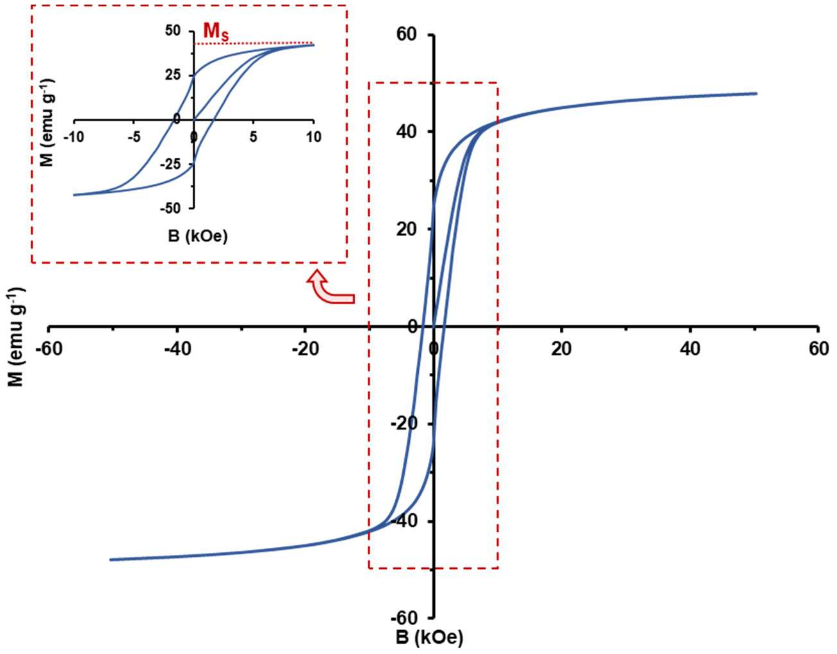

Fig. S3. Magnetization curve of the $CoFe_2O_4$@SiO_2@MPS@MAA-co-EGDMA sorbent.

Zeta potential – point of zero charge

Concerning the surface charge, Fig. S4 shows the zeta potential versus pH plot for a suspension of sorbent in different 10 mM o-phosphoric acid solutions adjusted to different pH values (i.e., 2.5, 2.8, 3.1, 3.6, and 4.0) with sodium hydroxide 1M. The responses at pH < 2.5 were not satisfactory due to the high conductivity of the sample at this pH. According to the obtained curve, the pH_{pzc} was determined to be ca. 3.3.

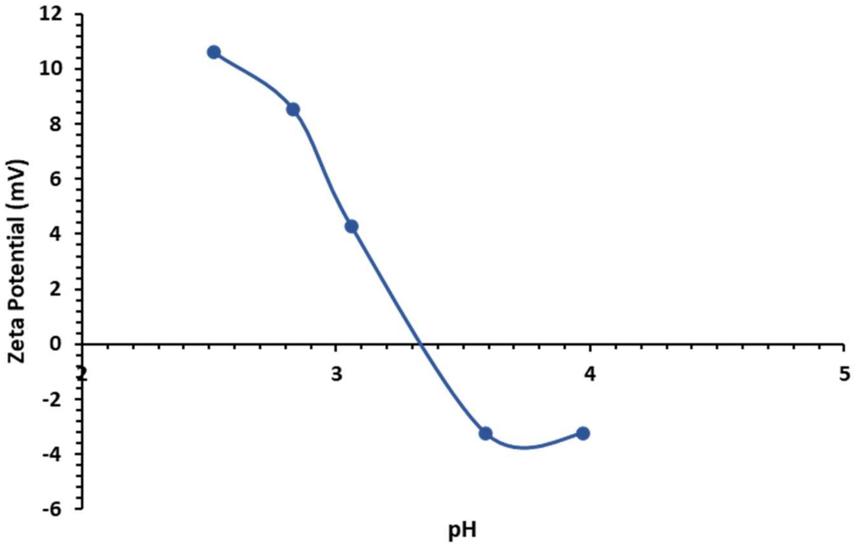

Fig. S4. Zeta potential of $CoFe_2O_4$@SiO_2@MPS@MAA-co-EGDMA sorbent.

Morphology

Morphology of $CoFe_2O_4$ MNPs, $CoFe_2O_4$@SiO_2, $CoFe_2O_4$@SiO_2@MPS, and $CoFe_2O_4$@SiO_2@MPS@MAA-co-EGDMA sorbent were determined by SEM operating at 10 kV. Representative SEM micrographs of these materials are shown in Fig. S5. As shown in these images, all the materials presented the expected spherical shape due to the successive coating of the $CoFe_2O_4$ MNPs (Fig. S5a) by silica (Fig. S5b), by MPS (Fig. S5c), and by the MAA-co-EGDMA polymer (Fig. S5d).

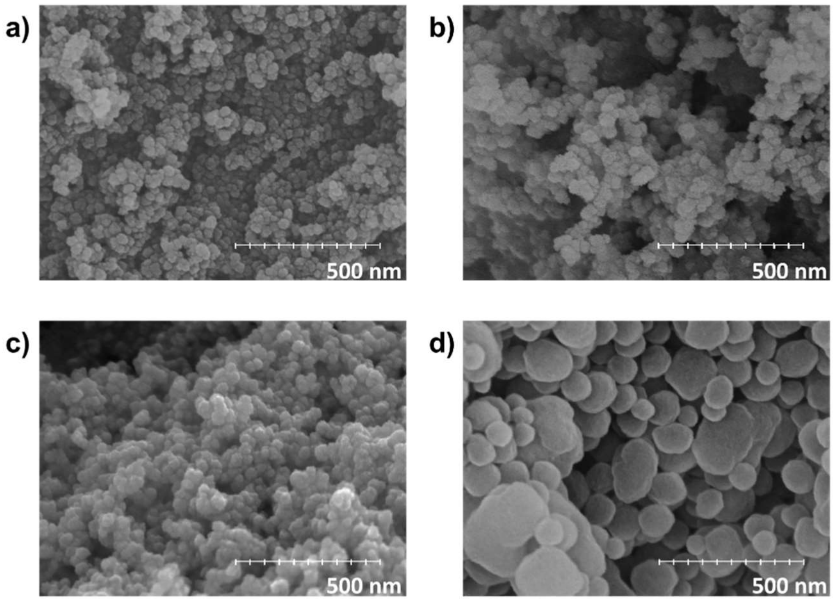

Fig. S5. SEM micrographs of the (a) $CoFe_2O_4$ MNPs, (b) $CoFe_2O_4$@SiO_2, (c) $CoFe_2O_4$@SiO_2@MPS, and (d) $CoFe_2O_4$@SiO_2@MPS@MAA-co-EGDMA sorbent.

Adsorption properties

A study of the adsorption capacity of the material was performed. To this respect, the adsorption-desorption isotherms and the specific surface area were measured. Most of the isotherms can be classified into six groups (i.e., types I-VI adsorption isotherms). When adsorbate-adsorbent interactions are higher than adsorbate-adsorbate interactions, type I, II, IV and VI isotherms are obtained. In this sense, the obtained curve (see Fig. S6) shows a Type-II isotherm, and the Brunauer-Emmett-Teller (BET) surface area was 60.7 ± 0.2 m^2 g^{-1}.

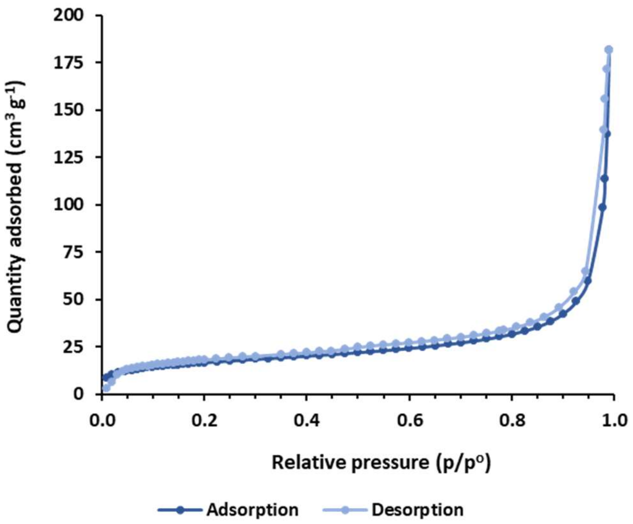

Fig. S6. Isotherm linear plot of the $CoFe_2O_4$@SiO_2@MPS@MAA-co-EGDMA sorbent.

Thermogravimetric analysis

Finally, Fig. S7 shows the TGA curve of the $CoFe_2O_4$@SiO_2@MPS@MAA-co-EGDMA sorbent under O_2 atmosphere. Two jumps of weight loss are observed in the thermogravimetric curve: the first one is observed from 25 to 100 ºC (6.0%) and it corresponds to the evaporation of adsorbed solvent or water, and the second one is observed from 300 to 400 ºC (53%) and it is attributed to the combustion of the organic coating (i.e., MPS and MAA-co-EGDMA). Therefore, from the thermogravimetric analysis it can be concluded that the sorbent is stable until 300 ºC.

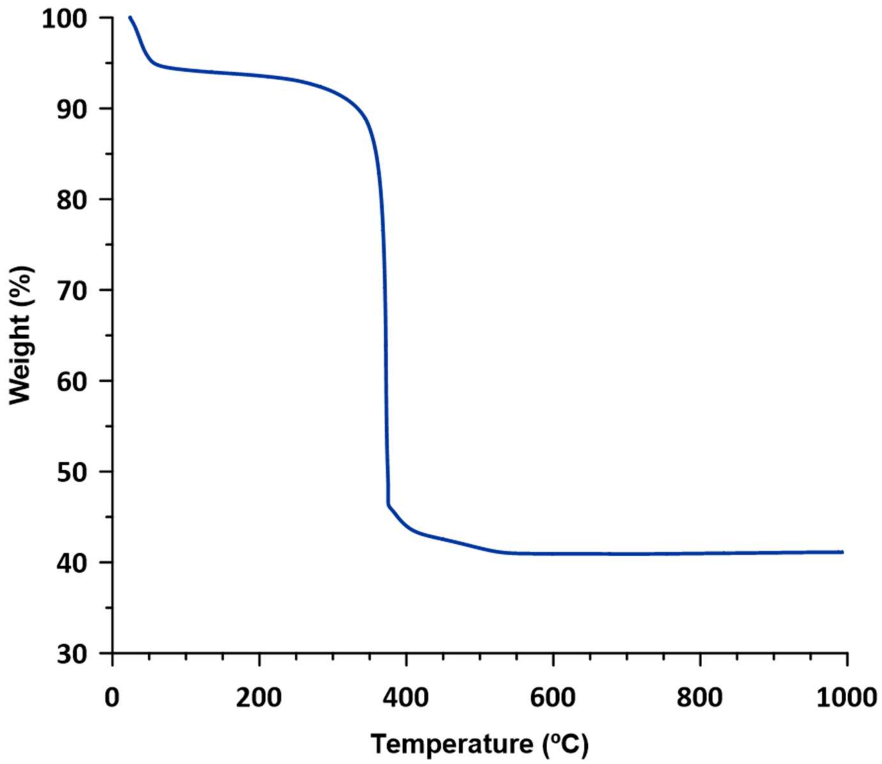

Fig. S7. TGA plot of $CoFe_2O_4$@SiO_2@MPS@MAA-co-EGDMA sorbent.

Box-Behnken design

The Box-Behnken designs are a class of rotatable or nearly rotatable second-order designs based on three-level incomplete factorial designs. The number of experiments required for the development of the designs (N) is defined as follows:

$$N=2k(k-1)+C_P \quad (2)$$

where k is the number of factors (i.e., 4); and C_P is the replicates of the central point. In this sense, according to Equation (2), 27 experiments were required, selecting 3 replicates of the central point. The range of each factor (Table S3) was defined between a high and a low value.

The following quadratic polynomial equation was applied to evaluate the multiple linear regression for each response:

$$Y=\beta_o+\sum \beta_i X_i+\sum \beta_{ii} X_i^2+\sum \beta_{ij} X_i X_j+\varepsilon \quad (3)$$

where Y is the analytical response; β_o is a constant term; β_i, β_j, and β_{ij} are the regression coefficients of the design; X_i and Xj are the different variables; and ε is the residual error.

The global desirability of the experiment (D) was applied to evaluate the different responses and to achieve the optimal extraction conditions to maximize the analytical responses of the analytes:

$$D=\left(d_1(Y_1)\cdot d_2(Y_2)\cdots d_n(Y_n)\right)^{1/n} \quad (4)$$

where n is the number of responses in the optimization process, and $d_i(Y_i)$ is the individual desirability of each response in the experiment. The individual desirability is calculated as:

$$d_i=\frac{Y_i-Y_{min}}{Y_{max}-Y_{min}} \quad (5)$$

Undesirable responses and fully desirable responses are ranged between 0 and 1, respectively, for each response.

Table S3. Box-Behnken Design for multivariate optimization of the critical variables.

Step	Sorbent amount (mg)		Extraction time (min)		pH		Ionic strength (%NaCl, w/v)	
	Uncoded	Coded	Uncoded	Coded	Uncoded	Coded	Uncoded	Coded
1	5	-1	5	-1	6	0	5	0
2	20	1	5	-1	6	0	5	0
3	5	-1	30	1	6	0	5	0
4	20	1	30	1	6	0	5	0
5	12.5	0	17.5	0	2	-1	0	-1
6	12.5	0	17.5	0	10	1	0	-1
7	12.5	0	17.5	0	2	-1	10	1
8	12.5	0	17.5	0	10	1	10	1
9	5	-1	17.5	0	6	0	0	-1
10	20	1	17.5	0	6	0	0	-1
11	5	-1	17.5	0	6	0	10	1
12	20	1	17.5	0	6	0	10	1
13	12.5	0	5	-1	2	-1	5	0
14	12.5	0	30	1	2	-1	5	0
15	12.5	0	5	-1	10	1	5	0
16	12.5	0	30	1	10	1	5	0
17	5	-1	17.5	0	2	-1	5	0
18	20	1	17.5	0	2	-1	5	0
19	5	-1	17.5	0	10	1	5	0
20	20	1	17.5	0	10	1	5	0
21	12.5	0	5	-1	6	0	0	-1
22	12.5	0	30	1	6	0	0	-1
23	12.5	0	5	-1	6	0	10	1
24	12.5	0	30	1	6	0	10	1
25	12.5	0	17.5	0	6	0	5	0
26	12.5	0	17.5	0	6	0	5	0
27	12.5	0	17.5	0	6	0	5	0

Optimization of the extraction variables

Analysis of variance (ANOVA) for each analyte was performed in order to assess the significance and adequacy of the model at a confidence level of 95%. In this sense, high coefficient of determination (88.9 – 96.0%) and good adjusted determination coefficient (76.0 – 91.4%) were accomplished, showing satisfactory correlation between the data obtained and the statistical model. Regarding the p-values, it can be inferred that the extraction time, and both the pH and the ionic strength of the donor phase, are factors that significantly affect the extraction of the analytes ($p < 0.05$). Moreover, the quadratic interaction sorbent amount/sorbent amount, sorbent amount/extraction time, sorbent amount/ionic strength, extraction time/pH of the donor phase, pH of the donor phase/pH of the donor phase, and ionic strength/ionic strength are also factors that affect the extraction ($p < 0.05$). The correlation of the different variables that affect the extraction of the target analytes are shown in Fig. S8.

According to the RSM curves shown in Fig. S9, optimum conditions in terms of desirability (estimated as described above) were achieved when using 15-20 mg of sorbent, likely due to the good dispersion of the material in the donor phase and the satisfactory elution of the analytes in the 0.5 mL of the desorption solvent. Since there were no significant differences between using 15 or 20 mg of sorbent, 15 mg was selected as optimum amount of sorbent. Regarding the extraction time, 5 min was enough to successfully carry out the extraction of the target analytes, and thus it was selected as optimum extraction time. In the case of the pH of the donor phase, although the best results were achieved at pH 6.2, there was no significant difference between 5 and 7 pH values. This behavior is likely due to the electrostatic interaction of the positive charge of the amine group of the TCAs (pKa 9.2 – 10.4) and the negative surface charge of the material (pH_{pzc} 3.3) provided by deprotonated carboxylic group of MAA. Therefore, the pH would not be essential to adjust given that the pH of diluted urine is nearby 6. Finally, the optimum ionic strength was achieved at 6.8% w/v NaCl. The increase of the ionic strength decreased the solubility of the TCAs in the aqueous donor phase (salting-out effect), improving the extraction. However, when an excess of salt is added, the viscosity of the donor phase increases, impairing the mass transfer of the analytes from the donor phase to the sorbent.

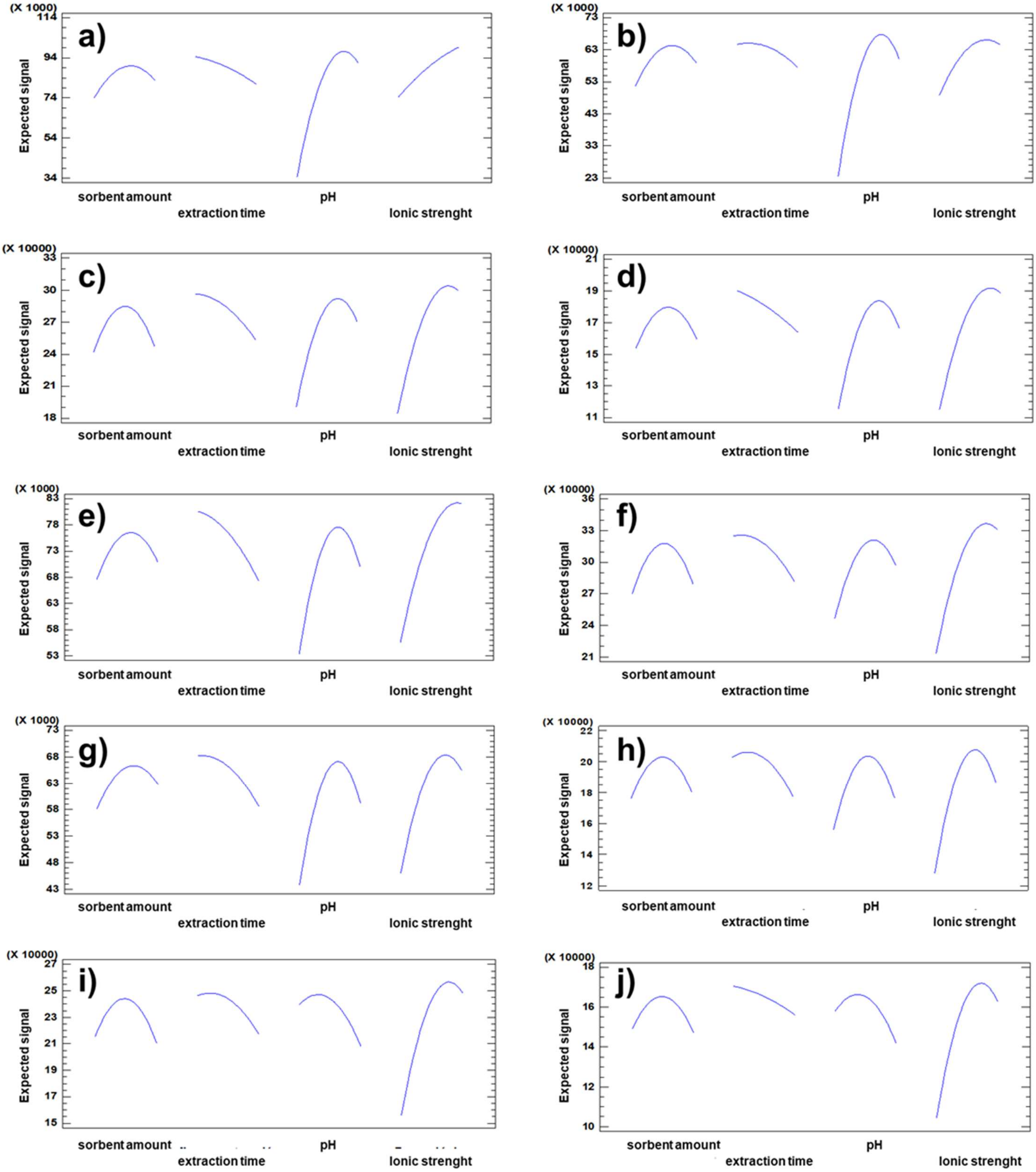

Fig. S8. Correlation of the different variables that potentially affect the extraction of (a) DOX, (b) NDOX, (c) IMP, (d) DIMP, (e) AMT, (f) TMP, (g) NORT, (h) NTMP, (i) CMP, and (j) NCMP.

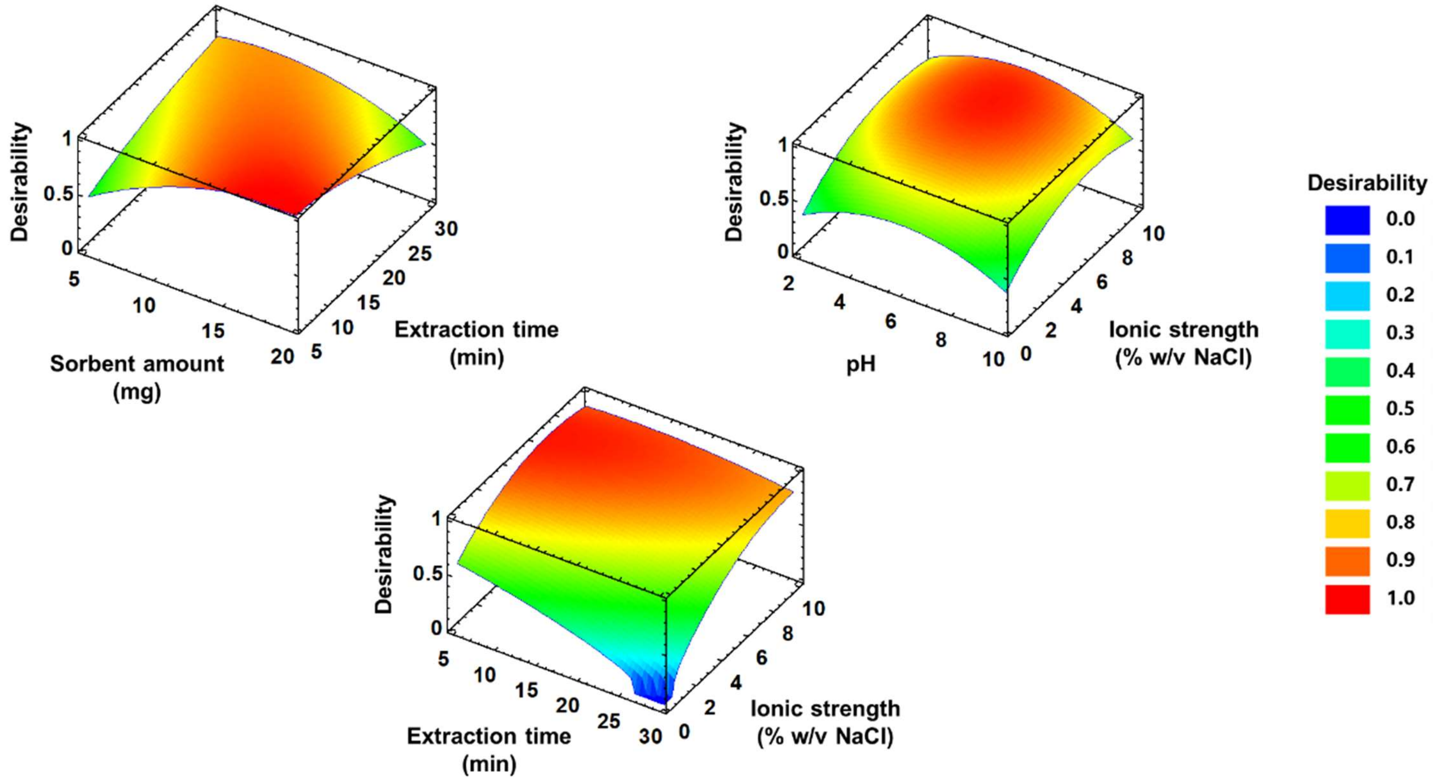

Fig. S9. Response surface of the desirability function representing the relation between the different variables affecting the extraction: (a) sorbent amount vs extraction time, (b) pH vs ionic strength, and (c) extraction time vs ionic strength.

Optimization of the desorption variables

Desorption solvent

After the extraction procedure, the coated stir bars containing the extracted analytes were immersed for 5 min in 0.5 mL of an appropriate solvent to elute them from the donor phase. In this sense, mixtures of MeOH and H_2O, without and with acetic acid or ammonia, were studied. It should be pointed out that previous experiments revealed that at least 30% of water was needed in the desorption solvent in order to avoid the peak splitting of the most polar TCAs (i.e., DOX and NDOX). As can be seen in Fig. S10, the best EF values were achieved when a mixture of MeOH:H_2O:AcOH 6:3:1 v/v/v was used. This is likely due to the breakdown of the electrostatic interaction and the subsequent electrostatic repulsion between the positively charged surface of the sorbent (pH_{pzc} of 3.3) and the positively charged amine groups of the TCAs (pKa 9.2 – 10.4) at this pH level (ca. 2.2). On the other hand, the mixture of MeOH:H_2O:NH_3 6:3:1 v/v/v also provided better results than MeOH:H_2O 6:4 v/v but in a lesser extent than MeOH:H_2O:AcOH 6:3:1 v/v/v, since just the breakdown of the electrostatic interaction was achieved.

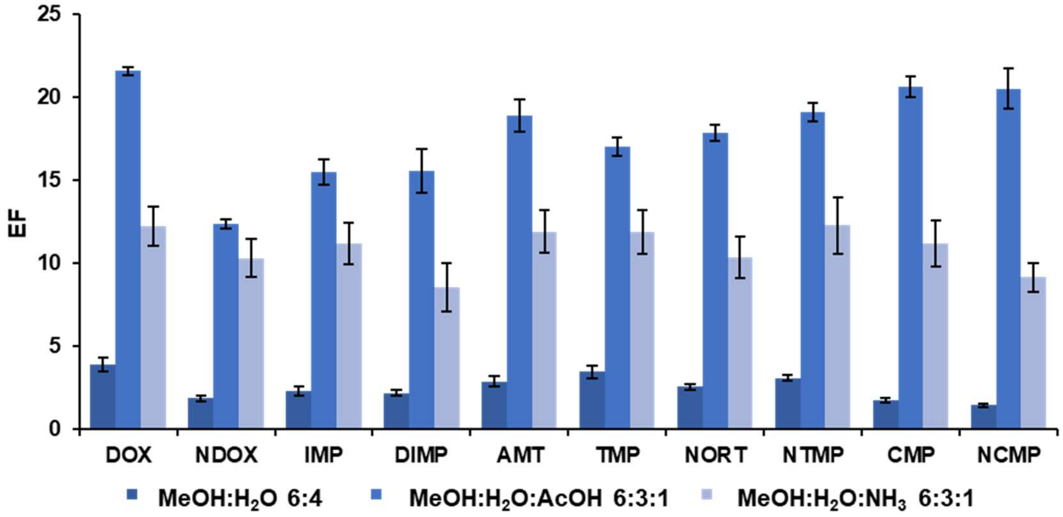

Fig. S10. Effect of the desorption solvent. Extraction conditions: sorbent amount, 15 mg; extraction time, 5 min; pH, not adjusted; ionic strength, 6.8% w/v NaCl. Desorption conditions: desorption time, 5 min; desorption volume, 0.5 mL.

Desorption time

In order to study the desorption time, the coated stir bars were immersed in 0.5 mL of MeOH:H_2O:AcOH 6:3:1 v/v/v after the extraction process was completed. The contact time between the sorbent and the desorption solvent must be enough to reach a quantitative desorption. Thereby, different desorption times (i.e., 1-10 min) were tested. The results (Fig. S11) show that the greatest EFs were accomplished between 5 and 10 min. Given that there were no great differences between both values, 5 min was selected as desorption time for further experiments.

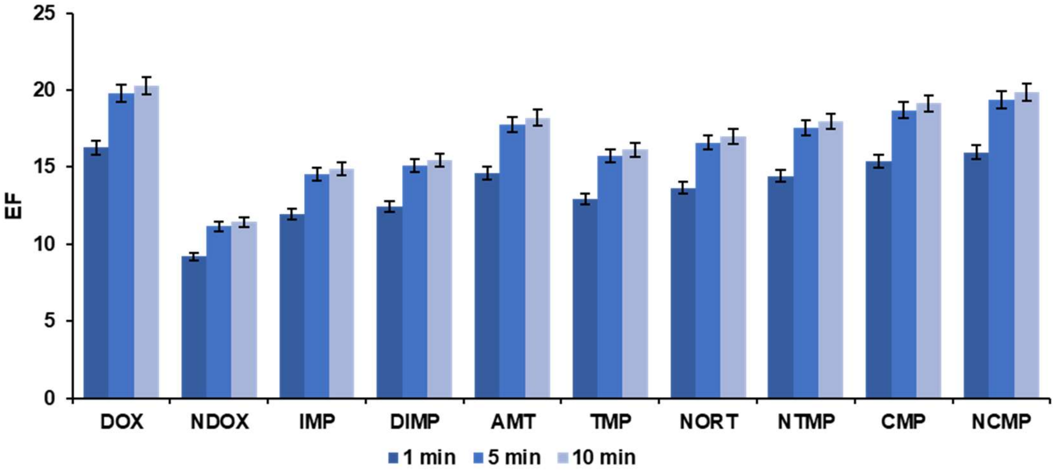

Fig. S11. Effect of the desorption solvent. Extraction conditions: sorbent amount, 15 mg; extraction time, 5 min; pH, not adjusted; ionic strength, 6.8% w/v NaCl. Desorption conditions: solvent desorption, MeOH:H_2O:AcOH 6:3:1 v/v/v; desorption volume, 0.5 mL.

Desorption volume

Finally, the desorption volume was studied by immersing the coated stir bars in 0.25 or 0.50 mL of MeOH:H_2O:AcOH 6:3:1 v/v/v for 5 min after the extraction process was completed. Lower desorption volumes did not cover the coated stir bar. As can be seen in Fig.S12, the best results were achieved when 0.25 mL of the desorption solvent was employed. As expected, a volume of 0.50 mL produced a dilution effect thus reducing the EF.

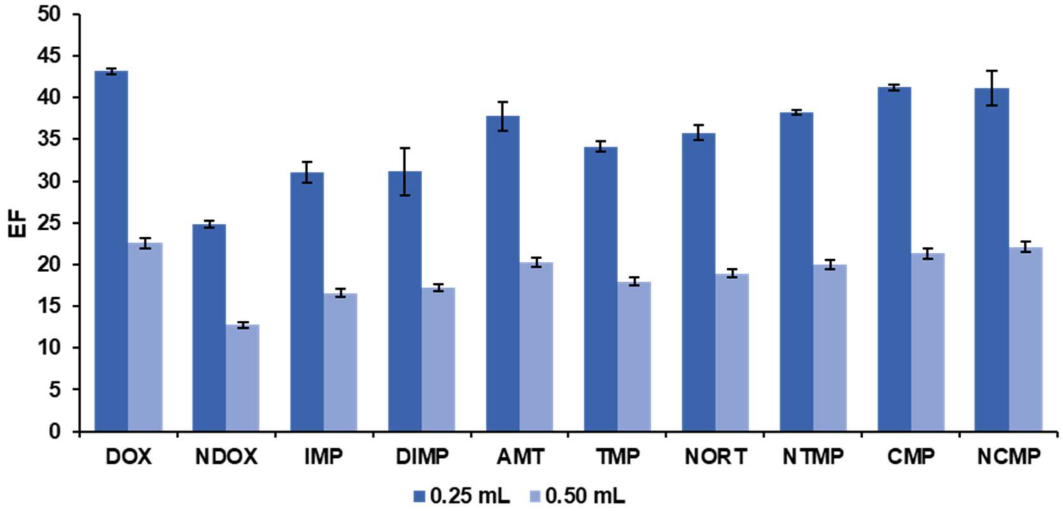

Fig. S12. Effect of the desorption solvent. Extraction conditions: sorbent amount, 15 mg; extraction time, 5 min; pH, not adjusted; ionic strength, 6.8% w/v NaCl. Desorption conditions: solvent desorption, MeOH:H_2O:AcOH 6:3:1 v/v/v; desorption time, 5 min.

Extraction efficiency of the $CoFe_2O_4$@SiO_2@MPS@MAA-co-EGDMA sorbent

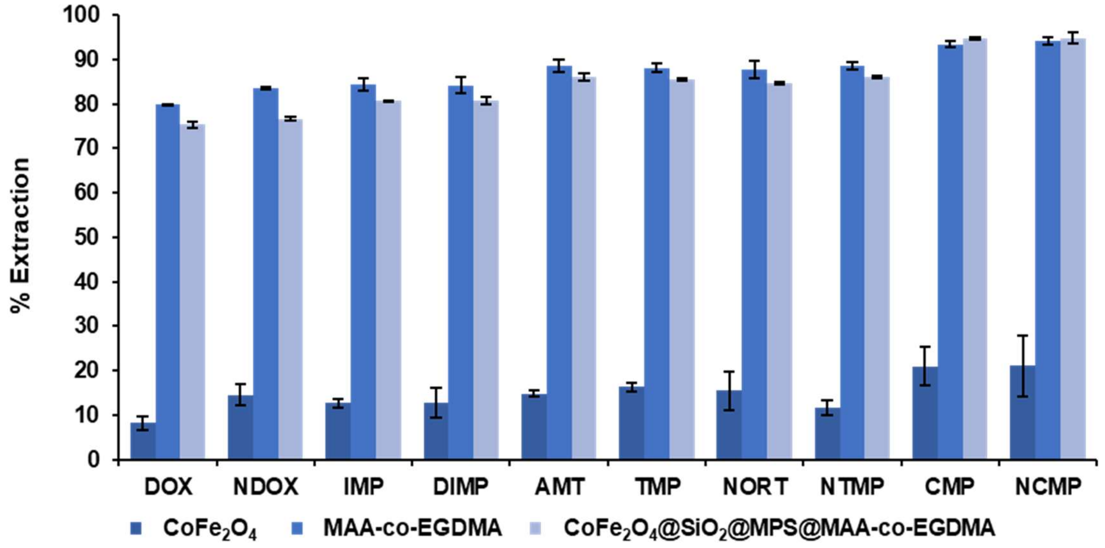

Fig. S13. Extraction efficiency of $CoFe_2O_4$ MNPs, MAA-co-EGDMA polymer, and $CoFe_2O_4$@SiO_2@MPS@MAA-co-EGDMA sorbent. Extraction conditions: sorbent amount, 15 mg; extraction time, 5 min; pH, not adjusted; ionic strength, 6.8% w/v NaCl.

Inter-batch repeatability of the $CoFe_2O_4$@SiO_2@MPS@MAA-co-EGDMA sorbent

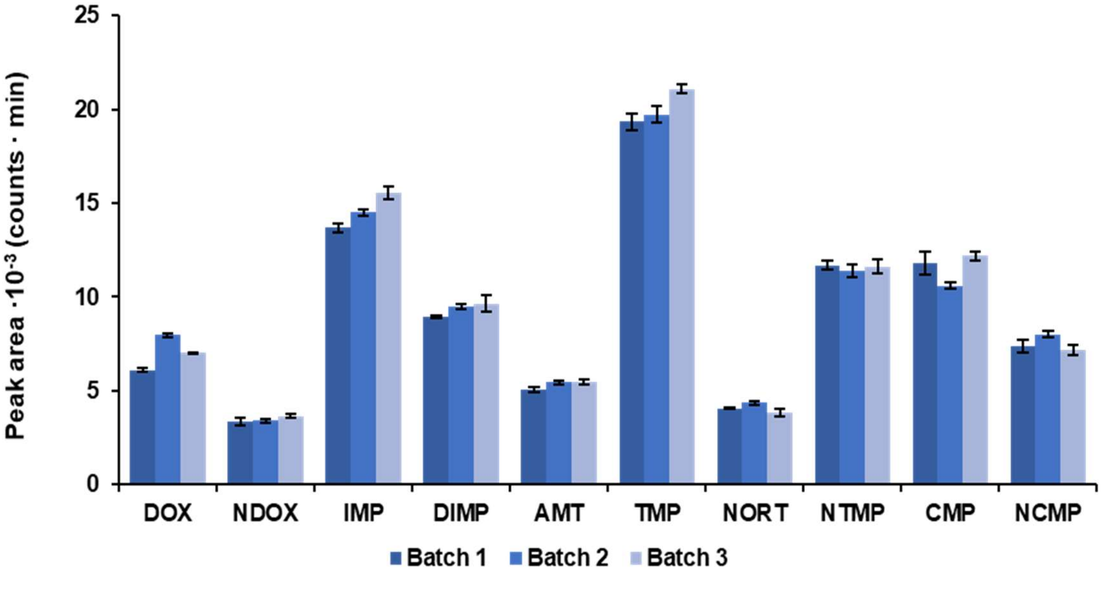

Fig. S14. Inter-batch repeatability of the $CoFe_2O_4$@SiO_2@MPS@MAA-co-EGDMA sorbent. Extraction conditions: sorbent amount, 15 mg; extraction time, 5 min; pH, not adjusted; ionic strength, 6.8% w/v NaCl. Desorption conditions: solvent desorption, MeOH:H_2O:HAcO 6:3:1 v/v/v; desorption time, 5 min; desorption volume, 0.25 mL.

Study of matrix effects

Table S4. Comparison between external calibration and matrix-matched calibration.

Compound	Standard matrix	Slope ± Deviation [a] (mL ng^{-1})	Student's t test [b]	
			t_{exp}	Equality
DOX	Water	24.3 ± 0.5	-	-
	Urine pool	15.5 ± 0.3	15.09	No
NDOX	Water	16.5 ± 0.3	-	-
	Urine pool	12.6 ± 0.5	6.69	No
IMP	Water	78.8 ± 0.9	-	-
	Urine pool	44.3 ± 0.9	27.11	No
DIMP	Water	44.2 ± 0.6	-	-
	Urine pool	29.8 ± 0.6	16.97	No
AMT	Water	25.8 ± 0.7	-	-
	Urine pool	13.2 ± 0.7	12.73	No
TMP	Water	85.8 ± 1.9	-	-
	Urine pool	50.4 ± 0.9	16.84	No
NORT	Water	17.7 ± 0.4	-	-
	Urine pool	10.5 ± 0.4	12.73	No
NTMP	Water	51.1 ± 0.9	-	-
	Urine pool	31.3 ± 1.2	13.20	No
CMP	Water	47.7 ± 1.9	-	-
	Urine pool	33.7 ± 1.6	5.64	No
NCMP	Water	32.8 ± 0.5	-	-
	Urine pool	22.1 ± 0.5	15.13	No

[a] Concentration range: 0.1 – 0.5 ng mL^{-1}. Number of calibration points: 5.

[b] Student's t-test for slopes comparison between aqueous and analyte-free urine pool (t_{crit}(0.05,8)=2.31

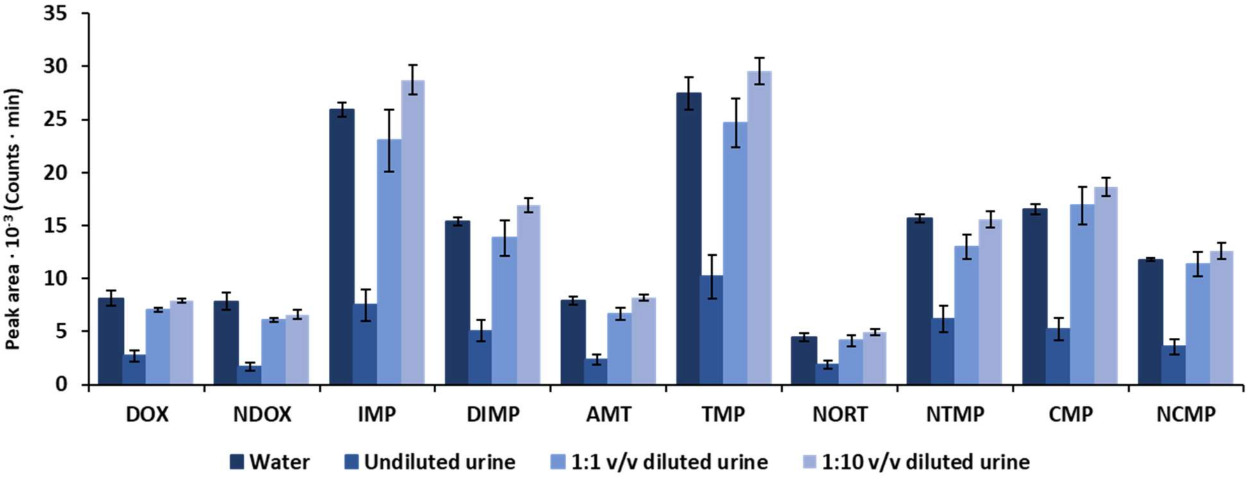

Fig. S15. Effect of the urine dilution on the analytical peak area of target analytes. Extraction conditions: sorbent amount, 15 mg; extraction time, 5 min; pH, not adjusted; ionic strength, 6.8% w/v NaCl. Desorption conditions: solvent desorption, MeOH:H_2O:HAcO 6:3:1 v/v/v; desorption time, 5 min; desorption volume, 0.25 mL. (All solutions were spiked at the same concentration level)
